# White matter brain structure predicts language performance and learning success

**DOI:** 10.1002/hbm.26132

**Published:** 2022-11-18

**Authors:** Stella M. Sánchez, Helmut Schmidt, Guillermo Gallardo, Alfred Anwander, Jens Brauer, Angela D. Friederici, Thomas R. Knösche

**Affiliations:** ^1^ Consejo Nacional de Investigaciones Científicas y Técnicas (CONICET) Buenos Aires Argentina; ^2^ Max Planck Institute for Human Cognitive and Brain Sciences, Brain Networks Group Leipzig Germany; ^3^ Laureate Institute for Brain Research Tulsa Oklahoma USA; ^4^ Institute of Computer Science Czech Academy of Sciences Prague Czech Republic; ^5^ Department of Neuropsychology Max Planck Institute for Human Cognitive and Brain Sciences Leipzig Germany; ^6^ Friedrich Schiller University, Office of the Vice‐President for Young Researchers Jena Germany; ^7^ Institute of Biomedical Engineering and Informatics TU Ilmenau Ilmenau Germany

**Keywords:** Cognitive performance, Language performance, Learning process, White matter, Working memory

## Abstract

Individual differences in the ability to process language have long been discussed. Much of the neural basis of these, however, is yet unknown. Here we investigated the relationship between long‐range white matter connectivity of the brain, as revealed by diffusion tractography, and the ability to process syntactically complex sentences in the participants' native language as well as the improvement thereof by multiday training. We identified specific network motifs by singular value decomposition that indeed related white matter structural connectivity to individual language processing performance. First, for two such motifs, one in the left and one in the right hemisphere, their individual prevalence significantly predicted the individual language performance, suggesting an anatomical predisposition for the individual ability to process syntactically complex sentences. Both motifs comprise a number of cortical regions, but seem to be dominated by areas known for the involvement in working memory rather than the classical language network itself. Second, we identified another left hemispheric network motif, whose change of prevalence over the training period significantly correlated with the individual change in performance, thus reflecting training induced white matter plasticity. This motif comprises diverse cortical areas including regions known for their involvement in language processing, working memory and motor functions. The present findings suggest that individual differences in language processing and learning can be explained, in part, by individual differences in the brain's white matter structure. Brain structure may be a crucial factor to be considered when discussing variations in human cognitive performance, more generally.

## Graphical abstract

Participants improve on processing syntactically complex sentences after intense training. Left and right hemisphere subnetworks predicted initial language performance. White matter tracts of a left hemisphere subnetwork changed during intense training. Initial performance is associated with the working memory system. Changes in individual performance over training depend on white matter reorganization.

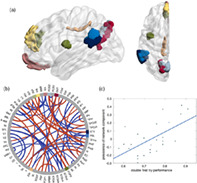

## 1 INTRODUCTION

Language is a cognitive domain that is considered specifically human, in particular when it comes to processing syntactically complex structures (Berwick & Chomsky, 2016; Fitch & Hauser, 2004; Hauser et al., 2002). Humans, however, differ in how well they deal with processing complex sentences, even in their native language, depending on their working memory capacity (Caplan & Waters, 1999; MacDonald et al., 1992). When this capacity is low, processing of ambiguous sentences becomes difficult (Fiebach et al., 2004; Friederici et al., 1998; Just & Carpenter, 1992). Independently, it has been shown that one's capability to process complex sentences in the native language can be improved in relatively short time by intense training, even in adults (Wang et al., 2021). This raises two questions: What is the neurobiological basis of behavioral differences in processing complex sentences, and what is the neural basis responsible for training‐induced performance improvements in processing such sentences?

The neuroanatomical and physiological underpinnings of language processing and language learning are certainly diverse. Language processing in the adult brain is mainly based on a left hemispheric network involving particular frontal, temporal, and parietal regions (for a review, see Friederici, 2011). By contrast, language learning—at least second language learning in adults—appears to involve additional brain regions (for a review, see Li et al., 2014). The multiple spatially separated regions involved in language processing are connected by dorsally and ventrally located long‐range fiber bundles running through the white matter of the brain (for an overview, see Friederici, 2017). Among these, the dorsal fiber tract targeting Broca's area is particularly crucial for the processing of syntactically complex sentences (Skeide et al., 2016; Wilson et al., 2011). The structural properties of these and other white matter fiber tracts are prime candidates for the explanation of interindividual differences in language performance and the effects of training during learning.

These properties include, but are not limited to, the trajectories and density of nerve fibers, determining which neurons may exchange information, as well as diameters and myelination of axons, impacting transmission speed as well as synchronization and ephaptic coupling between axons (Schmidt et al., 2021; Schmidt & Knösche, 2019). In living human brains, they are only indirectly accessible by imaging techniques, mainly based on magnetic resonance imaging (MRI). Diffusion weighted MRI (DWI) protocols most reliably deliver information on the spatial trajectories of nerve fibers (see Jones et al., 2013, for a critical review), although they may also be sensitive to other properties of the fibers, such as axonal diameter (Assaf et al., 2008; but see also Paquette et al., 2020) and myelin sheath thickness (g‐ratio; see Mohammadi & Callaghan, 2021).

Prior work has demonstrated white matter plasticity in multiple studies on second language learning. Many of them are cross‐sectional and compare populations with and without certain second language skills (Cummine & Boliek, 2013; Hämäläinen et al., 2017; Mamiya et al., 2016; Pliatsikas & Chondrogianni, 2015; Vandermosten et al., 2015). These studies therefore target white matter plasticity occurring over a long (and not precisely defined) period of time. The same applies to studies comparing populations with different first languages. In contrast, Schlegel et al. (2012) used diffusion MRI to show reorganization of major white matter fiber tracts over a period of 9 months, during which the participants intensively learned a second language (Chinese). Likewise, Flöel et al. (2009) used a region‐of‐interest approach focused on Broca's area and its right hemispheric homologue to identify changes in white matter as a function of artificial grammar learning at the group level.

Although the neural bases of first and second language acquisition are discussed to be partly overlapping (Perani & Abutalebi, 2005), there may be substantial differences, in that second language acquisition relies on more variable and widespread neural networks (Cargnelutti et al., 2019; Dehaene et al., 1997) compared to the relatively well defined first language network (Friederici, 2011, 2017). For native language acquisition, we found developmental changes in the gray and white matter of the language network (Cafiero et al., 2019; Ekerdt et al., 2020; Huber et al., 2018). In adults, the language capabilities are largely established, which is paralleled by a fully matured language network (Skeide et al., 2016). However, adults may be trained to further improve in certain aspects of their mother tongue, and the question is whether this leads to noninvasively detectable reorganization of the white matter. Changes related to learning rate in language relevant gray and white matter regions were observed in adults as a function of word learning in their native language over a short period of time (<1 h) (Hofstetter et al., 2017). Also for other domains of learning, relatively short‐term (from hours to weeks) reorganizations of white matter have been observed at the group level, such as computer gaming (Hofstetter et al., 2013), juggling (Scholz et al., 2009) and balancing (Taubert et al., 2010), tactile training based on Braille reading (Debowska et al., 2016), and mental complex multiplications (Klein et al., 2019). Nevertheless, it is still open whether individual differences in native language processing and learning relate to individual white matter brain structure. Moreover, it remains undetermined whether training in structural aspects of language beyond mere word acquisition (thematic role assignment, syntax processing) will lead to structural changes in the white matter that can be detected noninvasively.

In order to find answers to these questions, we need to utilize language material that is syntactically demanding enough to provide room for training‐induced improvement even in adult native speakers. In a previous study (Wang et al., 2021), we have shown that center embedded German sentences do indeed fulfill this need, as German native speakers improved their performance in understanding thematic role assignments during a 4‐day training period. While, both, single and double embedded sentences were tested, the effect was most pronounced for the double embeddings, while for the single embeddings the performance was already very high at the beginning of the training (ceiling effect).

Therefore, we investigate whether the behavioral differences in adults processing double center embedded sentences in their native language are rooted in structural differences in white matter fiber connections, whether the success of intense training over a relatively short period of time is predicted by such structural traits, and whether such training would in fact induce further structural changes. For this, we used behavioral (performance) data from our previous experiment (Wang et al., 2021). We explored and compared structural connectivity matrices obtained by tractography from diffusion MRI data acquired before and after training and related these to behavioral performance at both time points. In the analyses, we focused on the aspect of white matter fiber connectivity in the language network, rather than metrics that might be more sensitive to local microstructural properties, such as fractional anisotropy (FA) or mean diffusivity (MD).

## 2 METHODOLOGY

### 2.1 Paradigm

The experiment was designed to investigate how brain function and white matter connectivity change during multiday language training. Training was performed during four out of the five working days of 1 week. On the first training day, prior to the experiment, reading span (Daneman & Carpenter, 1980) and digit span (WAIS‐IV, Wechsler, 2008) were acquired as measures for language specific and general working memory abilities, respectively. On each training day, the participants listened to 66 German center‐embedded sentences: half with single and the other half with double center embedding.

Example for single center embedding:

*Ihr Freund sagte*, *dass Gustav*, *der Marlene überschätzte*, *Klavier spielt*, *um sich zu bilden*.

*[Her friend said that Gustav*, *who overestimated Marlene*, *plays piano*, *in order to educate himself*.*]*

Example for double center embedding:

*Yvonne dachte nicht*, *dass Bernd*, *der Leo*, *der intelligent ist*, *liebte*, *Maria verfolgen will*.

*[Yvonne did not think that Bernd*, *who loved Leo*, *who is intelligent*, *wants to pursue Maria*.*]*

Each sentence was followed by a content question probing the participant's understanding of the thematic role assignment. The answer was recorded by delayed key press within a predefined time window and acknowledged by a visual feedback (smiley/frowny). In case of a wrong or missing answer, the same sentence was repeated and additionally displayed on screen. A different content question was then asked and again acknowledged by feedback. Irrespective of the correctness of the second answer, the experiment was continued with the next trial. Trials with single and double embedding were randomly intermixed. The experiment was divided into 3 blocks, each of which contained 22 sentences and lasted about 7 min. Between the blocks, participants enjoyed a short break. During the measurements, MEG was recorded with a Neuromag Vectorview device (results reported in Wang et al., 2021).

The performance of the participants was measured as the percentage of correct answers to the first content questions (#correct/(#correct + #incorrect + #missed)).

Finally, T1, diffusion, and resting‐state functional (not reported here) MRI data were acquired three times: before the experiment (scan a), immediately after it (scan b), and 3 weeks later (scan c). See Section 2.3 for technical details.

### 2.2 Participants

The sample included 28 right‐handed participants and inclusion criteria were as follows: subjects were 18–35 years old at the time of recruitment, they were German native speakers with normal or corrected to normal hearing and vision, and no history of substance abuse (alcohol or drugs). Subjects with neurological or psychiatric disorders, past neurosurgery, neuroactive medication, claustrophobia, pregnancy, or other contraindications for MRI were excluded. Written informed consent was obtained from all participants prior to the experiment. The study was approved by the ethics committee of the University of Leipzig.

### 2.3 MRI data acquisition

MR images were acquired with a 3 T Siemens Magnetom Prisma MRI scanner. A high‐resolution (1 mm^3^) structural T1‐weighted scan was obtained (MP‐Rage, TR = 1.3 s, TE = 3.93 ms; *α* = 10°; 1 × 1 × 1 mm^3^). DWI was acquired with the standard GE‐EPI protocol. The employed parameters for diffusion data were: TR = 12 s, TE = 100 ms, A/P phase encoding direction, 72 slices, FOV = 220 × 220 mm^2^, acquisition matrix 128 × 128, 1.7 mm^3^ isotropic voxels, 60 diffusion‐weighted images (*b* = 1000 s/mm^2^), and 7 no diffusion weighting (*b*_0_) images. Multiband and fat saturation techniques were implemented to improve data quality.

### 2.4 Atlas selection and DWI preprocessing

In this study, we used the HCP‐MMP1 atlas (Glasser et al., 2016), which includes 180 cortical regions per hemisphere. This freely available atlas has been created by a sophisticated machine learning approach. It combines information about cortical architecture, function, connectivity, and topography in a precisely aligned group average of 210 brains, and has been carefully cross‐validated. It is therefore arguably one of the most comprehensive and reliable human brain atlases available today. After executing an intrasubject cross‐modal registration, based on a rigid body transformation, we performed a projection of this atlas onto each participant's MNI‐T1 image. Therefore, the outcome for each subject was the HCP‐MMP1 atlas co‐registered with diffusion data. In addition, in order to avoid spurious interhemispheric connections, we added to the analysis a five‐region‐parcellation of the corpus callosum from a white matter parcellation. This step was executed using the Freesurfer software.

After visual inspection for large artifacts, diffusion data were corrected from susceptibility‐induced distortion, subject motion, and artifacts due to eddy currents with standard procedures from the FMRIB Software Library [FSL, https://fsl.fmrib.ox.ac.uk/fsl/fslwiki/FSL (Jenkinson et al., 2012)] as was applied in previous works (Cummine & Boliek, 2013; Neef et al., 2018; Salminen et al., 2016). Following the same preprocessing implemented in Neef et al. (2018), to estimate diffusion parameters at each voxel we applied Bayesian inference through the tool *bedpostx*, which also resolves voxels with crossing fibers. Subsequently, probabilistic tractography was applied to reconstruct sample streamlines using the *probtrackx2* command with default parameters (5000 samples per seed voxel, maximum of 2000 steps per streamline, curvature threshold of 0.2, step length of 0.5 mm). As was explained by Assaf et al. (2017), the reconstruction of commissural pathways by a tractography algorithm presents several limitations due to their complex white matter architecture, involving crossing, fanning and kissing fibers. Therefore, in order to avoid this limitation when tracking fibers through the highly convergent corpus callosum area (but not missing these fibers), we performed the tractography procedure separately for each hemisphere adding the five parcels of the corpus callosum in the list of target regions. This way, we generated a seed‐to‐seed connectivity matrix per subject and hemisphere. The matrix entry *C*_*ij*_ corresponds to the number of streamlines generated from region *i* (source) and entering region *j* (target). Both tools, *bedpostx* and *probtrackx2*, are part of the FSL software.

### 2.5 Analysis of connectivity

Based on the connectivity matrices described above, and the two main questions raised in the Introduction, we investigated the following relations:

The first main question concerns the relation between the brain structural precondition and performance prior to training.

(Q1) Does the a priori connectivity (scan a, before training) correlate with the initial performance (day 1)?

(Q2) Does this a priori connectivity (scan a) correlate with the performance change over the 4‐day‐experiment (day 4 minus day 1, training effect)?

The second main question concerns the relation between brain structural changes and training induced performance change with two sub‐questions:

(Q3) Does the change in connectivity over the experiment (the difference in connectivity values, scan b—scan a) correlate with the performance change over the 4‐day‐experiment (training effect, day 4–day 1)?

(Q4) Although we focus on the correlation between performance and connectivity across individuals, we will nonetheless ask whether there are group‐level changes in connectivity between the time points before and after the 4 days of training (scans b vs. a), as well as 3 weeks after the training (scans c vs. a).

Because the behavioral training effect was most prominent for the double center embedded sentences (see Section 3), we used those trials to quantify performance (initial and change over training period) as described in Section 2.1.

As test quantities for the connectivity, we chose the prevalence of certain network motifs in each subject, hemisphere, and scan, as derived from singular value decomposition (SVD). It has been applied to functional connectivity derived from fMRI (Worsley et al., 2005) or EEG (Rubega et al., 2019), and more generally to large data sets, such as DNA microarray data (Liu et al., 2003; Wall et al., 2003) or image processing (Chowdhary & Acharjya, 2018). This technique identifies a number of ordered connectivity motifs with the following properties: (1) they are mutually orthogonal (i.e., linearly independent); (2) by weighted summation, they reproduce each individual connectivity matrix (per scan and subject); and (3) they are chosen such that the first motif explains the maximally possible proportion of the variance across all connectivity matrices, the next motif explains the maximum of the remaining variance, and so forth. Note that each of these motifs necessarily contains positive and negative entries (due to the orthogonality condition). This means that a higher prevalence of a motif in a particular connectivity matrix (as quantified by its weight) means that some connections are increased and others decreased.

Separately for the two hemispheres, the connectivity matrices (as described in Section 2.4) of all subjects and scans were arranged as column vectors, forming a matrix *M* which was decomposed into left singular vectors *U*, singular values ∑, and right singular vectors *V*: *M* = *U*∑*V* ^*T*^. The left singular vectors *U* represent the orthogonal and normalized network motifs, the singular values ∑ indicate their prevalence across all structural matrices (explained variance), and the right singular vectors *V* indicate the relative prevalence (weight) of each network motif in each subject and scan. The number of motifs equals the rank of *M*, which is the product of the numbers of subjects and scans, thus 28 for Q1‐2 (only scan a used) and 56 for Q3 (scans a and b used). Each column of the *V* matrix can now be used to perform the statistical tests and correlation analyses described above for the corresponding network motif (corresponding column of *U*). If, for example, these weights show a significant correlation with the initial performance of the participants, this shows that the respective motif as a whole predicts the performance, while separate statements about each single connection cannot be made. In this respect, the method is akin to the region‐of‐interest approach, but with two important differences. First, the SVD approach identifies motifs as collections of connections (“regions of interest”), which are not restricted to spatially compact regions and are identified from the data according to their joint impact onto the variability across subjects and scans. Second, within each motif, different connections have different weights (including negative ones), which quantify how much each connection contributes to that motif. A statistical effect for one motif can therefore be interpreted as mainly stemming from its largest contributors.

This network‐based analysis avoids the massive need for correction that would occur when testing each of the about 16,000 connections separately. Also the analysis of classical voxel‐wise diffusion metrics, like FA or MD, would cause similar multiple testing problems, besides being less specific with respect to network connectivity, which is the main target of our investigation. Our choice is further motivated by the notion that in the brain network nodes and connections do not act in isolation, but as part of larger (sub‐)networks. Consequently, any kind of specialization, either occurring during a lifetime development (relevant for Q1, Q2) or induced by an intense training process (relevant for Q3, Q4) should also involve coherent changes of entire network patterns, or motifs.

For each of the above questions (Q1–Q4), we performed statistical tests. Since the performance and connectivity values cannot be guaranteed to be normally distributed, we exclusively used non‐parametric statistical tests. Specifically, we applied Spearman's correlation with a significance level of 0.05, corrected for multiple comparisons by Bonferroni correction (correcting for the number of motifs; 28 for Q1 and Q2, and 56 for Q3). For the pairwise comparison in Q4, we performed Mann–Whitney *U* tests, also followed by Bonferroni correction (correcting for the number of connections, ~16,000).

Connectivity matrices, and relevant MATLAB code used for SVD analysis and plotting are available on a GitHub repository at https://github.com/hschmidt82/Yerevan_public.

## 3 RESULTS

### 3.1 Behavioral

**FIGURE 1:**
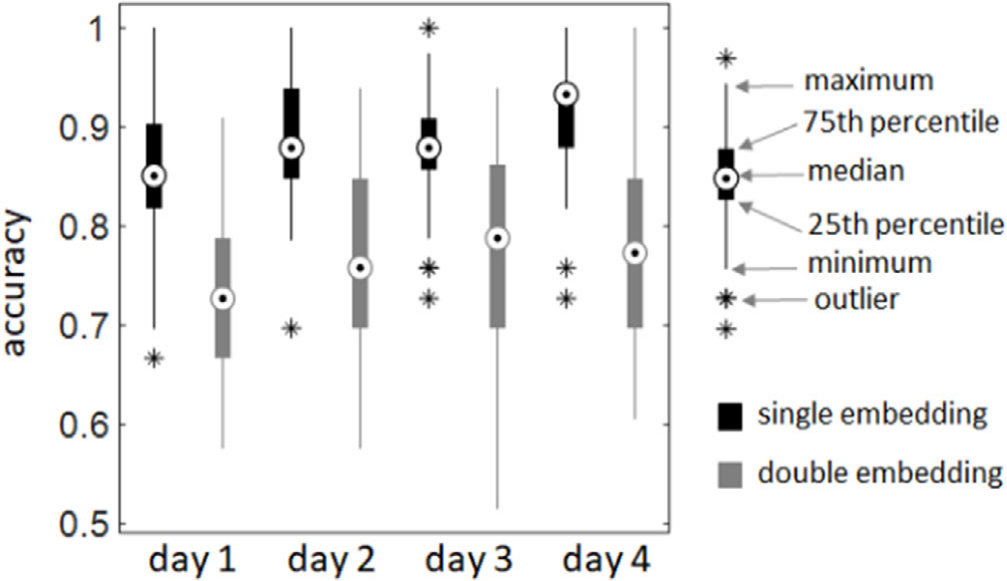
*Behavioral data*. For both, single and double embedding, there is a significant difference between days 4 and 1 (*p* < .005, corrected). For the double embeddings, there are also significant differences between days 1 and 2 as well as between days 1 and 3 (*p* < .05, corrected). Adapted from Wang et al. (2021)

Figure 1 displays the mean performances over subjects for each training day, separately for single and double embedding sentences. These results, which were already reported and discussed elsewhere (Wang et al., 2021), show a significant performance improvement for both types of sentences, but for the simpler sentences this improvement had to be small since the initial performance was already quite high. Due to this ceiling effect, we decided to use for the connectivity analyses the double embedded sentences only.

In order to probe how the initial performance was related to the participants' memory abilities, we correlated it with reading span and digit span (levels reached, forward and backward averaged). Reading span showed a significant correlation with the performance on single (*r* = .31, *p* < .05), but not on double embedded sentences (where performance was initially quite low). Digit span did not yield any significant correlation.

### 3.2 Left hemisphere

**FIGURE 2:**
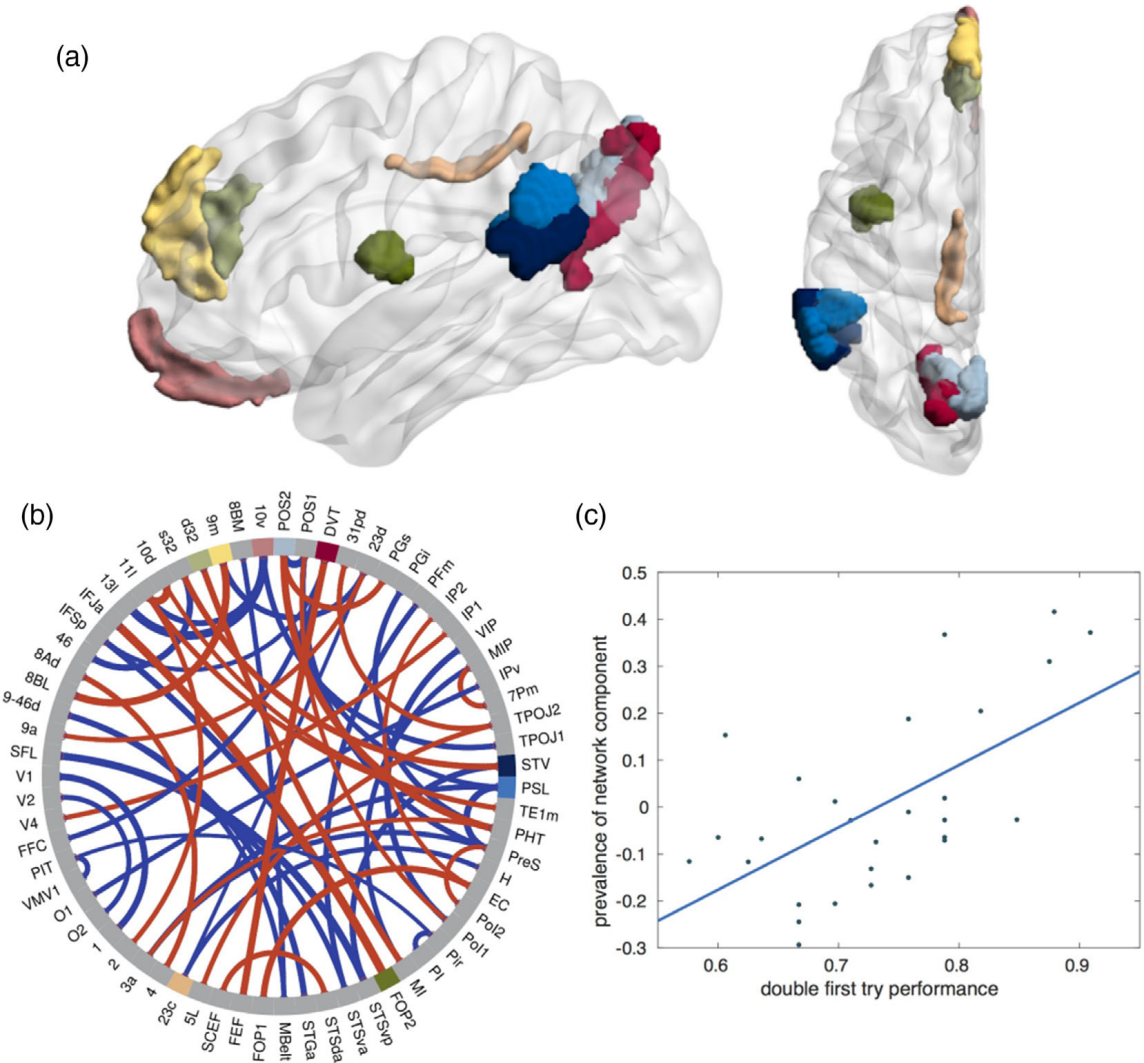
*Network motif 1 in the left hemisphere*, *whose prevalence before the training correlates with the baseline performance on day 1*. (a) Sagittal and axial view (created using BrainNet viewer; Xia et al., 2013) of nine brain areas where the network component has highest (absolute) connectivity (labels according to Glasser et al., 2016, in panel b). (b) Chord plot of strongest connections in the network motif, with line thickness indicating the absolute weight of the connection within the motif and the color indicating the sign (red: positive, blue: negative). Note that connections that have negative weightings in the network motif actually correlate negatively with the motif prevalence. Then, 67 of the 185 brain areas are plotted, the main constituents of the network motif (panel a) are highlighted in color. (c) Regression plot of network prevalence before the training (right singular vector V) against the performance of subjects during the first day of training on the double center embedding task (*r* = .567, *p* = .046)

The network‐based analysis yielded two relevant network motifs related to behavioral performance. The first motif appears to exhibit a predisposition effect, as its prevalence in the subjects before the experiment (scan a) correlates with their performance on the first day (see Figure 2c). This gives a direct answer to question Q1. Figure 2a,b displays the main areas and connections of this network component. It is composed of white matter fiber tracts, which most strongly connect areas in the medial prefrontal cortex (10v, 9m, d32), the posterior cingulate cortex (23c, dorsal visual transition area [DVT], parietal occipital sulcus [POS2]), the frontal operculum (FOP2), and the temporo‐parieto‐occipital junction (TPOJ1; perisylvian language area [PSL], superior temporal visual area [STV]). While some of these areas are known for their specific involvement in language processing (particularly PSL), others have been reported to be activated in theory of mind (watching socially interacting objects; areas 10v, 9m, PSL, STV), motor functions (9m, 23c, FOP2, POS2, PSL, STV, DVT), and working memory tasks (9m [faces], d32 [all images], 23c [body], DVT [places], POS2 [body, faces]) (Glasser et al., 2016). Some of these areas are mainly involved in connections that correlate negatively with the first‐day performance (23c, PSL, 10v).

**FIGURE 3:**
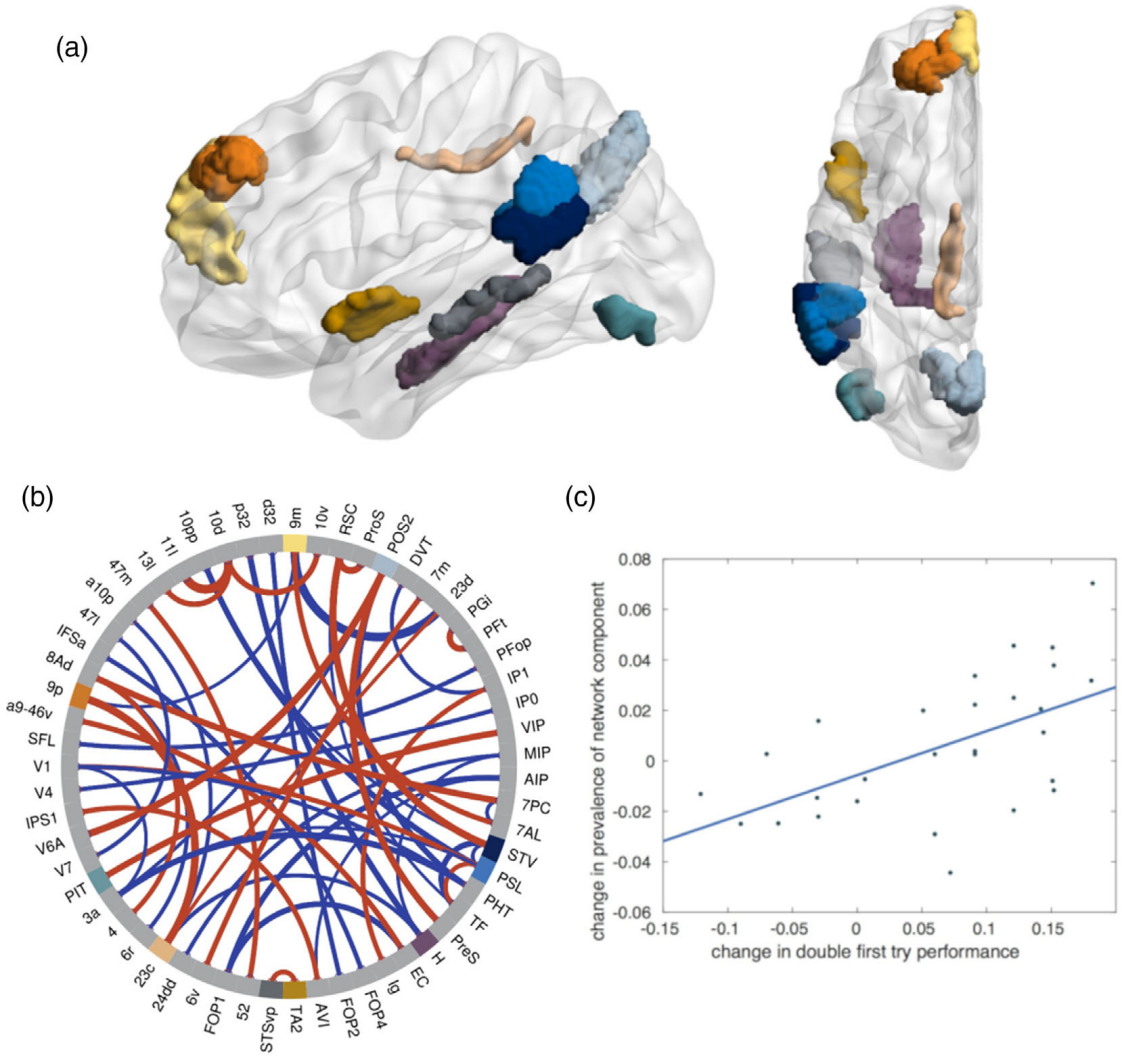
*Network motif 2 in the left hemisphere*, *whose changes over the training period correlate with the performance change between days 1 and 4*. (a) Sagittal and axial view (created using BrainNet viewer; Xia et al., 2013) of 10 brain areas where the network component has highest (absolute) connectivity (labels according to Glasser et al., 2016, in panel b). (b) Chord plot of strongest connections in the network motif, with line thickness indicating the absolute weight of the connection within the motif and the color indicating the sign (red: positive, blue: negative). Note that connections that have negative weightings in the network motif actually correlate negatively with the motif prevalence. Then, 59 of the 185 brain areas are plotted, the main constituents of the network motif (panel a) are highlighted in color. (c) Regression plot of change in network prevalence between scans before and after training (right singular vector V) against change in the performance of subjects between days 1 and 4 of training on the double center embedding task (*r* = .606, *p* = .035).

The second motif indicates structural changes related to the training process. Its difference in prevalence between scan b (after training) and scan a (before training) significantly correlates with the change in performance between the last (day 4) and the first (day 1) days of training (Figure 3). This finding directly relates to question Q3. Figure 3 shows the main areas and connections of this network component. They partially overlap with the first network motif (areas 9m, 23c, POS2, STV, and PSL). In addition, this network motif includes white matter fiber tracts connecting parts of auditory association cortex (TA2 on planum polare, STSvp in superior temporal sulcus), dorsolateral prefrontal cortex (9p), hippocampus (H), and ventral stream visual cortex (posterior inferior temporal [PIT]). Again, these areas have been reported in a wide variety of experimental conditions, including language processing (PSL, TA2, STSvp, H); theory of mind (watching socially interacting objects; areas 9m, 9p, PSL, STV, STSvp); motor functions (9m, 23c, POS2, PSL, STV, STSvp); and working memory tasks (9m [faces], 23c [body], POS2 [body, faces], H [all], PIT [faces]) (Glasser et al., 2016). Again, some of the areas are mainly involved in connections that correlate negatively with the performance change (PSL, H).

None of the two left hemispheric motifs yielded significant results concerning question Q2 (prediction of training effect by pre‐experimental connectivity) and Q4 (group level change of connectivity through training). Moreover, none of the motifs significantly correlated with the individual reading span or digit span.

### 3.3 Right hemisphere

**FIGURE 4:**
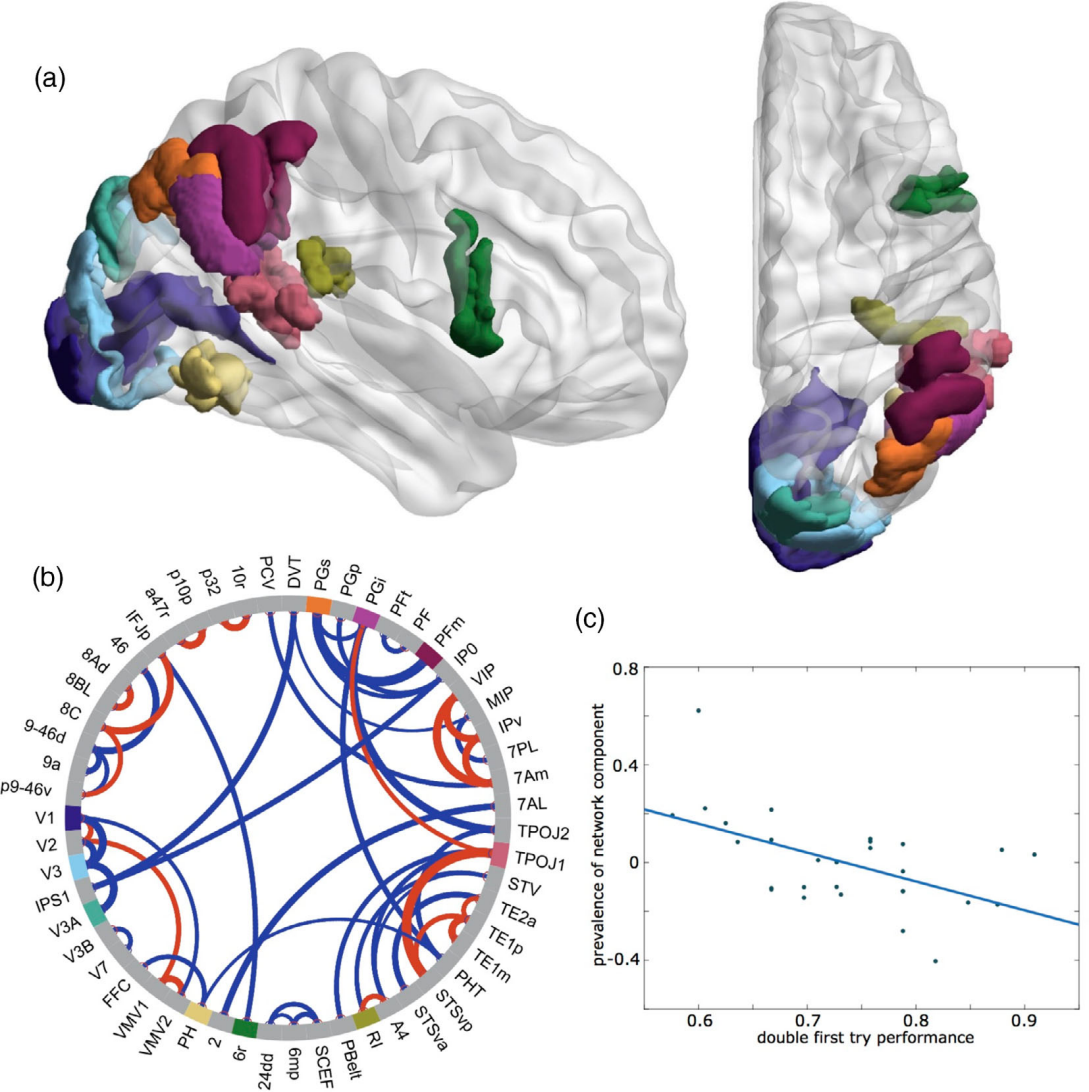
*Network motif 3 in the right hemisphere*, *whose prevalence before the training correlates with the baseline performance on day 1*. (a) Sagittal and axial view (created using BrainNet viewer; Xia et al., 2013) of nine brain areas where the network component has highest (absolute) connectivity (labels according to Glasser et al., 2016, in panel b). (b) Chord plot of strongest connections in the network motif, with line thickness indicating the absolute weight of the connection within the motif and the color indicating the sign (red: positive, blue: negative). Note that connections that have negative weightings in the network motif actually correlate negatively with the motif prevalence (and therefore positively with the performance, see panel c). Then, 55 of the 185 brain areas are plotted, the main constituents of the network motif (panel a) are highlighted in color. (c) Regression plot of network prevalence before the training (right singular vector V) against the performance of subjects during the first day of training on the double center embedding task (*r* = −.575, *p* = .038)

The network‐based analysis did not yield any structural changes related to the training process (Q2–Q4). However, a motif was identified whose prevalence correlates with the subjects' performance on the first day, thus relating to question Q1. Figure 4 displays the main areas and connections of this network component. This network motif appears very different from the left‐hemisphere motifs 1 and 2, and mainly comprises white matter connections of the inferior parietal cortex (PGs, PGi, PFm), the TPOJ1, primary auditory cortex (retro‐insular [RI]), premotor cortex (6r), and visual cortex (V1, V3, V3A, PH). While for some of the inferior parietal areas, a specific involvement in language processing has been reported (PGi, TPOJ1), others are known to be specifically deactivated for language (PGs, PFm) (Glasser et al., 2016). Interestingly, the most strongly represented part of the primary auditory cortex, area RI, is reported as deactivated during language processing, while the other core and belt areas are strongly activated in the same task (Glasser et al., 2016, figure 12). Some of these areas are mainly involved in connections that correlate negatively with the first‐day performance (TPOJ1, RI). Note that because the entire motif shows a negative correlation (Figure 4c), these are connections with a positive weighting, marked in red in Figure 4b.

Moreover, the motif showed no significant correlation with the individual reading span or digit span.

## 4 DISCUSSION

In this study, our goal was to elucidate the relationship between individual white matter brain anatomy, as revealed by diffusion tractography, and the comprehension of syntactically complex sentences in adult native speakers. We were asking if and how the given structure of the white matter of an individual influences their ability to extract thematic roles from center embedded sentences (Q1), and how it affects the improvement of that ability through intense training (Q2). Moreover, we also sought to answer the question whether intense training can induce measurable white matter changes, that is white matter structural plasticity. Specifically, we tested if such changes are related to the individual performance improvement (Q3), and if there was a general (group‐level) difference in white matter connectivity between the time points before and after training (Q4). Each of these questions was approached based on the prevalence of network motifs (or subnetworks).

We were able to identify three network motifs, two of which (one in each hemisphere) predicted the initial performance of the participants, while the third motif (in the left hemisphere) changed during training in correlation with the individual performance change.

We will now discuss these data in more detail. The first main question concerns the relevance of the individual preexisting white matter connectivity for language performance and training success in the native language. Here, we can conclude that there are indeed global structural properties of the white matter connectome, which predict the individual performance in comprehending complex sentences, and these properties influence diffusion tractography (Q1). In the left hemisphere, the identified network motif (Figure 2) is widely spread over the cortex and involves connections between areas that are known to be engaged in a variety of different brain functions. It contains connections that correlate positively or negatively with the performance, respectively. Positive correlations were mainly found for fiber tracts connecting areas known for their involvement in working memory tasks (9m, d32, DVT, POS2), while some areas related to language and theory of mind tend to have many connections that correlate negatively with the individual performance (PSL, 10v). This suggests that the different initial performances of the participants (67…100% for single embedded sentences and 58…91% for double embedded sentences) are rooted to some extent in differences in their working memory system. This presumption is further supported by the finding that the initial performance on single embedded sentences, which was already quite high prior to training in most subjects, correlated with the individual reading span score. The fact that reading span, but not digit span, showed such correlation might hint that language specific rather than general working memory is relevant here. These interpretations are interesting given that psycholinguistic theory has proposed that the ability to deal with syntactically complex sentences is influenced by the individual working memory capacity (Just & Carpenter, 1992; MacDonald et al., 1992).

In addition, there was also a right hemispheric network motif, the prevalence of which predicted the individual performance before the training (Figure 4). In contrast to the motif in the left hemisphere, the right hemispheric motif had a strong focus on connections among modality‐specific cortices (visual, auditory, motor), and additionally involved many working memory specific areas (PGs, PFm, 6r). Again, connections of areas that are known to be involved in language tasks (TPOJ1) tend to correlate negatively with performance, suggesting that increased performance in our task does not rely on language specific brain circuits.

These very same network motifs (or any other network motif), however, did not predict the ability of the participants to improve their performance through training (Q2). Naturally, it must remain open whether this is because the individual training abilities are governed by other factors than those reflected by white matter tractography, or because the statistical power for the correlation with training induced performance improvement was not sufficient.

Remarkably, the network motifs found in this study did not exhibit any prominent involvement of the syntax‐related Broca's area (areas 44 and 45 in the Glasser atlas), which has been strongly associated with syntax processing in the language network (Friederici, 2017; Wu et al., 2019). This is somewhat in contrast to previous work on structural connectivity: White matter connections between area 44 and the posterior temporal cortex were shown to correlate with performance on processing complex sentences during development (Skeide et al., 2016). In adults, the white matter connection with that area has been found to correlate with individual performance in an artificial grammar‐learning task using a region‐of‐interest approach (Flöel et al., 2009). Here, we find that individual performance differences of adults processing center embedding sentences of their native language mainly rest on differences in white matter connections among working memory‐related regions rather than syntax‐related regions. This suggests that, in contrast to children learning their first language and to adults engaged in a dedicated grammar learning task, performance improvement in our task relies more on improvements in working memory than in core syntactic processing. Note, however, that in our previous work on functional connectivity (Wang et al., 2021), we found (in the same participants) that BA 44 did exhibit training related changes in its neural activity for the double center embedding sentences. We also demonstrated strong functional interaction between BA 44 and both the inferior frontal sulcus and PGi (part of inferior parietal cortex), suggesting that BA 44 may interact with different types of working memory systems. Taken together, it appears that for the type of center embedding sentences we used in our study, training leads to changes in structural connectivity among areas relevant for working memory performance, which functionally interact with the more syntax‐specific network centered at BA 44.

The second main question investigated here focused on training and thereby learning‐related white matter plasticity. We could show that the individual performance change over the training period significantly correlated with the individual prevalence change of a particular network motif in the left hemisphere (Figure 3). This finding suggests that individual learning success for processing complex sentences depends on some reorganization of the white matter within the left hemisphere (Q3). At the group level, our analyses did not reveal significant structural changes in the white matter (Q4), although the participants significantly improved their performance at the group level (Figure 1). In other words, averaging across the individuals' white matter structure could not explain the observed group performance difference; rather it was the individual brain structure, which provided an explanation.

Interestingly, the motif related to individual performance change bears substantial similarity to the one that predicts the initial performance (see above), but also features some differences. The most striking difference is the additional involvement of temporal areas (TA2, STSvp; compare Figures 2a and 3a). Most of the strong connections show positive correlations with the training success, connecting areas reported for language (TA2, STSvp); theory of mind (9m, 9p, STV, STSvp); working memory (9m, 23c, POS2, PIT); and motor functions (9m, 23c, POS2, STV, STSvp). Prominent areas with connections showing negative correlations were PSL (involved in various functions, including language) and H (hippocampus). This picture seems to suggest that the training process is associated with some reorganization of widespread networks involved in multiple functional aspects of the brain.

Structural plasticity of the white matter due to short‐term language training has been demonstrated before by Hofstetter et al. (2017). Although they have not performed a connectivity analysis as we did in this study, they found differences between diffusion MRI metrics before and after the training in the white matter of inferior frontal gyrus, middle temporal gyrus, and inferior parietal lobule in the left hemisphere, which is in rough agreement with our results. Note, however, that their experiments just involved the acquisition of novel words (flower names) rather than processing complex sentence structures, as in our case.

From our results, it appears that there are certain structural properties of white matter fiber connections which are relevant for the processing of syntactically complex sentences and even exhibit some plasticity in response to training. These properties are captured by diffusion MRI and further influence tractography streamline counts. However, it is not possible to uniquely map those observations back onto particular microstructural properties. Tractography results, and diffusion MRI in general, are influenced by a multitude of properties, including myelination, fiber diameter, fiber density, variability of fiber orientation, glia cell density, and others. For a detailed treatment of this problem, see Jones et al. (2013). Given that we observe training‐induced plasticity, remodeling of myelination might be a strong candidate, as activity dependent myelination has been established in a number of in vivo and in vitro studies (for an overview, see, e.g., Chorghay et al., 2018).

## 5 CONCLUSION

In summary, we can state that the individual modulation of the ability to extract thematic roles from center‐embedded sentences depends on widespread networks of white matter fibers connecting areas in different parts of the cortex. The initial individual level of language performance, which has been acquired throughout life, seems to depend mainly on a network connecting (temporo‐)parietal, medial prefrontal, frontal opercular, and cingulate areas, many of which are known for their involvement in working memory abilities. The rapid performance improvement of processing syntactically complex sentences induced by the relatively short, but intense, training during the experiment appears to induce changes in a diverse network, additionally involving medial and superior temporal regions, spanning language, working memory, theory of mind, and motor functions. Taken together, this might suggest that under the pressure of the training, subjects used a multitude of strategies to improve their performance, but in the long run, working memory is the key ability to master complex center‐embedded sentences. In order to substantiate this conclusion, further dedicated studies will be necessary, involving appropriate control conditions to specify the importance of working memory for syntax processing.

The present data suggest that individual language performance can be explained by individual white matter structural patterns—a relation, which may hold for individual differences observed in cognitive functions more generally. Thus, the individual differences in the brain's white matter structure may be a crucial factor to be considered when discussing variations in cognitive performance.

## CONFLICT OF INTEREST

The authors declare no conflict of interest.

## Data Availability

Freesurfer and FSL open‐sourced softwares were implemented to process and analyze T1 and DWI data. Connectivity matrices, and relevant MATLAB's code used for SVD analysis and plotting are available on a GitHub repository at https://github.com/hschmidt82/Yerevan_public.
